# What affects functional ovarian reserve, thyroid function or thyroid autoimmunity?

**DOI:** 10.1186/s12958-016-0162-0

**Published:** 2016-05-10

**Authors:** Andrea Weghofer, David H. Barad, Sarah Darmon, Vitaly A. Kushnir, Norbert Gleicher

**Affiliations:** Department of Obstetrics and Gynecology, Medical University Vienna, Vienna, Austria; The Center for Human Reproduction, New York, NY USA; The Foundation for Reproductive Medicine, New York, NY USA; Department of Obstetrics and Gynecology, Albert Einstein College of Medicine, Bronx, NY USA; Department of Obstetrics and Gynecology, Wake Forest University, Winston Salem, NC USA; Laboratory for Stem Cell Biology and Molecular Embryology, The Rockefeller University, New York, NY USA

**Keywords:** Thyroid stimulating hormone (TSH), Infertility, Ovarian reserve, Thyroid function, Thyroid autoimmunity, Anti-Müllerian hormone (AMH)

## Abstract

**Background:**

Thyroid dysfunction is the most common autoimmune endocrine disorder in women of reproductive age, and is associated with menstrual irregularities, anovulation and infertility. Whether it is thyroid function or thyroid autoimmunity that affects functional ovarian reserve (FOR, i.e., the small growing ovarian follicle pool) reflected in anti-Müllerian hormone (AMH) has, however, remained under dispute.

**Methods:**

We investigated in 225 infertile women whether thyroid function, after adjustment for thyroid autoimmunity, affects FOR within what is considered normal thyroid function (TSH, 0.4–4.5μIU/mL) by assessing AMH levels in reference to TSH levels, stratified for TSH < or ≥3.0μIU/mL. Thyroid autoimmunity was defined by presence of anti-thyroid peroxidase, −thyroglobulin and/or -thyroid receptor antibodies.

**Results:**

Mean age of studied women was 38.4 ± 5.0 years; their mean AMH was 1.3 ± 2.0 ng/mL and mean TSH 1.8 ± 0.9 μIU/mL. Thyroid autoimmunity was present in 11.1 % of patients. Women with TSH <3.0μIU/mL presented with significantly higher AMH compared to those with TSH ≥3.0μIU/Ml (*P* = 0.03). This difference remained significant after adjustment for thyroid autoimmunity as well as age (*P* = 0.02).

**Conclusions:**

Even after adjustment for thyroid autoimmunity and age, TSH <3.0μIU/mL in euthyroid infertility patients is associated with significantly better FOR (higher AMH) than TSH ≥3.0μIU/mL. This observation suggests a direct beneficial effect of lower TSH levels on follicular recruitment, and warrants investigations of thyroxin supplementation in infertile women with TSH levels ≥3.0μIU/mL in attempts to improve FOR.

## Background

Thyroid dysfunction is the most common endocrine disorder in women of reproductive age. Overt and subclinical hypothyroidism may cause menstrual irregularities and anovulation [1], and has been associated with female infertility [2]. These observations have led to the commonly adopted clinical practice of supplementing women trying to conceive with thyroxin if their TSH levels are ≥2.5 μIU/mL [3]. Investigations of this TSH cut off resulted, however, in conflicting results [4–6]: Reh et al., reported comparable pregnancy and delivery rates in euthyroid in vitro fertilitzation (IVF) patients with TSH cut offs of 2.5 μIU/mL and 4.5 μIU/mL [7], while Murto et al. identified TSH <2.5 μIU/mL and anti-Müllerian hormone (AMH) ≥10pmol/L (1.4 ng/ml) as significant predictors of live births in women with unexplained infertility [8].

Based on such diverging findings, some suggested that a TSH 2.5 μIU/mL may be too low to impair reproductive function [9]; others suggested that, as estrogen concentrations rise during follicular development, more thyroxin is bound, leaving less free thyroid hormone available for clinical utilization. As a result, TSH levels increase [10, 11].

During controlled ovarian hyperstimulation (COH), TSH increases 50–80 % from cycle start to ovulation induction with human chorionic gonadotropin (hCG), though most patients will around ovulation still experience TSH concentrations < 2.5 μIU/mL [9, 12]. Those with initial TSH above 3.0 μIU/mL at cycle start will, however, likely experience significant clinical hypothyroidism.

How abnormal thyroid function affects female fertility remains unknown. Reported associations with menstrual irregularities and anovulation in hypothyroidism suggest that thyroid dysfunction might impair follicular growth and maturation [13]. Assuming this to be the case, TSH levels should influence AMH concentrations, independent of thyroid autoimmunity and female age. A recent report by Busnelli et al. supports such an assumption by demonstrating increasing TSH levels during COH, and a trend toward lower AMH concentrations in women with TSH levels ≥ 2.5μIU/mL seeking fertility treatments [12].

AMH is produced by small growing follicles, from primary up to small antral follicles and, therefore, reflects what is called functional ovarian reserve (FOR) [14]. If thyroid function affects follicular growth and development, higher AMH concentrations should be observed in women with lower TSH levels independent of thyroid autoimmunity and female age. This study was designed to investigate this hypothesis.

## Methods

### Study population

This study investigated 225 infertile women who between July 2009 and March 2014 underwent an initial work up prior to fertility treatments at the Center for Human Reproduction (CHR) in New York. Only women with normal TSH levels (i.e., TSH 0.4–4.5μIU/mL) were eligible for enrollment. As AMH levels decrease under oral contraceptives (OC) and during pregnancy [15, 16], pregnancy and OC usage up to three months prior to testing served as exclusion criteria.

### Laboratory assays

As part of routine pre-IVF evaluations, AMH, TSH and thyroid antibody status, i.e. anti-thyroid peroxidase (TPO), −thyroglobulin (TG) and –thyroid receptor antibodies are assessed. TSH assessments were made in blood serum, using standard commercial third generation electrochemiluminescence immunoassay, with reference values of 0.4–4.5 μIU/mL as normal range. This normal TSH range was subdivided into low- and high- normal ranges, based on TSH levels < or ≥ 3.0 μIU/mL. AMH levels were assessed in blood serum by a 2nd generation Beckman Coulter assay, with values being log-transformed to account for skew among study subjects. Thyroid autoantibodies were assessed in blood serum, utilizing electrochemiluminescence immunoassay.

### Ovarian stimulation

COH for IVF was strictly standardized, with only two ovarian stimulation protocols in use: Women with normal FOR (NFOR) under age 38 were stimulated in long agonist cycles with 150–300 IU of gonadotropins daily; while women above age 38 and with low FOR (LFOR) for age (defined as outside of the 95 % CI for age of FSH and/or AMH) were stimulated in a microdose agonist cycle with 450–600 IU of gonadotropins daily. Both study groups involved similar distributions of both stimulation protocols.

### Statistics

Low-normal and high-normal TSH was compared using a two-sampled t-test for continuous variables and a two-sided Fisher exact test for categorical variables. Since there was a statistical difference in AMH and thyroid autoimmunity between TSH classifications, a multiple logistic regression model was used, with AMH and thyroid autoimmunity predicting TSH classification. This model passed the Hosmer-Lemeshow goodness-of-fit test.

Analyses were conducted using SPSS 22.0 under the supervision of the center’s senior medical statistician (SD). A *P*-value <0.05 was considered statistically significant.

### Institutional review board

The presented data only involved retrospective review of medical records and data retrieval from an anonymized research database. Patients at CHR sign an informed consent at initial consultation, which allows for such reviews if the patient’s medical record remains confidential and her identity protected. These conditions were met in this case, allowing for expedited review and approval by the Institutional Review Board (IRB) of The Center for Human Reproduction, New York, NY.

## Results

Patient characteristics are presented in Table 1. Mean age for study participants was 38.4 ± 5.0 years. Mean AMH levels were 1.3 ± 2.0 ng/mL, mean TSH levels were 1.8 ± 0.9 μIU/mL. Body mass index (BMI) and ethnicity were comparable between TSH groups (*p* = 0.58 and *p* = 0.49). Thyroid autoimmunity was present in 11.1 % of all patients. TPO antibodies were positive in 11.1 % of patients and TG antibodies in 1.8 % of women. There were no patients with thyroid receptor antibodies. Patients with low-normal and high-normal TSH levels were of comparable age (i.e. 38.3 ± 5.1 vs. 38.9 ± 4.4 years, *P* = 0.67). Women with high-normal TSH levels presented with thyroid autoimmunity in 26.9 %, while thyroid autoimmunity was present in only 9.0 % of women with low-normal TSH levels (*P* = 0.01). Thyroid autoimmunity was mainly attributed to TPO antibodies (Table 1).

**Table 1:** Patient characteristics of 225 women who underwent work up for in vitro fertilization

	All (*n* = 225)	TSH < 3μIU/mL (*n* = 199)	TSH ≥ 3μIU/mL (*n* = 26)	*P*-value
Female age (years)	38.4 ± 5.0	38.3 ± 5.1	38.9 ± 4.4	0.67
TSH (μIU/mL)	1.8 ± 0.9	1.6 ± 0.6	3.5 ± 0.5	
Thyroid autoimmunity (%)	11.1 %	9.0 %	26.9 %	0.01
Thyroid peroxidase antibodies (%)	11.1 %	9.0 %	26.9 %	0.02
Thyroglobulin antibodies (%)	1.8 %	1.0 %	0.8 %	0.10
AMH (ng/mL)	1.3 ± 2.0	1.4 ± 2.0	0.8 ± 1.8	0.02
Positive Thyroid autoimmunity	0.9 ± 1.3	1.1 ± 1.5	0.5 ± 0.6	0.45
Negative Thyroid autoimmunity	1.4 ± 2.1	1.4 ± 2.1	0.9 ± 2.1	0.04

Values are presented as means ± standard deviation

Women with TSH <3.0 μIU/mL presented with significantly higher mean AMH levels (1.4 ± 2.0 ng/mL) than those with TSH ≥3.0 μIU/mL (0.8 ± 1.8 ng/mL; *P* = 0.02). Those findings remained significant when the analysis was adjusted for presence or absence of autoimmunity and age (*P* = 0.02) (Table 2).

**Table 2:** Logistic regression model for the probability of TSH ≥ 3 μIU/mL in 225 euthyroid infertile women

	Estimate	OR	*p*-value
Thyroid autoimmunity	1.23	3.41	0.02
log (AMH)	−0.40	0.67	0.03

Not surprisingly, thyroid autoimmunity was found to be a significant predictor of TSH classification. The estimated odds of a patient with positive thyroid autoimmunity exhibiting a high-normal TSH was 3.14 times that of a patient with negative thyroid autoimmunity and the same AMH level. Conversely, for a single unit log increase in AMH, odds of having a high-normal TSH level decreased by 33 % after controlling for thyroid autoimmunity.

## Discussion

Whether and, if so how, thyroid function affects FOR has remained unresolved. We in this study attempted to pierce out how thyroid function, thyroid autoimmunity and, possibly age, may interphase in answering this question. In this context it is important to note that this study was performed in women within euthyroid range to exclude secondary effects of cofounders that may be associated with abnormal thyroid function, like for example hyperprolactinemia.

Consequently, we here, even within normal thyroid function levels, are able to report a significant association of TSH levels with FOR, as assessed by AMH. What is generally considered sub-clinical hypothyroidism, reflected in TSH levels of ≥3.0μIU/mL, therefore, already appears to exert negative effects on FOR, resulting in significantly lower AMH levels. That this effect is thyroid function-dependent and not a consequence of thyroid autoimmunity was demonstrated in the study by the complete absence of any effects of adjustment for thyroid autoimmunity on the significance of the findings. Significance was also not affected by age, thus suggesting that here observed association of thyroid function with FOR holds at all ages. However, women included in our study had a mean age of 38.4 ± 5 years. It would therefore be interesting to see whether the lack of association can be confirmed in a study group of young women.

Here reported data are supported by a very recent publication by Kuroda et al., who also reported a clear statistical association between elevated TSH levels and decreases in AMH concentrations [17]. This Japanese study, however, investigated this association in infertile as well as fertile women, and unrestricted to the normal TSH range. Combined, both studies, thus, suggest that hypothyroidism already from very mild stages on in all women negatively affects FOR.

These findings explain why thyroid hormone supplementation has been reported to improve pregnancy potential in euthyroid women with high-normal TSH levels [12]. They, however, also contribute to the ongoing controversy in the medical literature whether thyroid function or thyroid autoimmunity are more important in affecting female reproduction [18]. Here presented data suggest that thyroid function appears to have the upper hand - at least when it comes to effects on FOR. One may, however, argue that thyroid autoimmunity has an impact on thyroid function, as tissue damage results in TSH level increases in women with thyroid autoantibodies [19]. Karmisholt et al. demonstrated highly significant associations between TPO-autoantibodies and TSH increase. They also reported that a considerable number of TPO positive women showed declining thyroid function over one year while none of the study subjects presented with improved TSH levels [20].

FOR is defined as the cohort of small growing follicles, consistently recruited from a woman’s primordial follicle pool. Though the follicular recruitment process in the human experience is not fully understood yet, the number of follicles recruited within a given time unit are believed to directly relate to the size of a woman’s primordial follicle pool [21]. As the granulosa cells of small growing follicles produce AMH, this hormone, except at extreme ages, is increasingly considered the most accurate marker of the growing follicle pool and, therefore, of ovarian function [14]. In general, higher AMH concentrations are associated with larger oocyte yields and improved pregnancy potential [22].

Our here reported results are indirectly also supported by previous reports on negative impact of hypothyroidism on prolactin, gonadotropin releasing hormone and sex steroid levels [23]. Moreover, the human ovary contains specific binding sites for thyroxin, which suggests direct effects of thyroid hormone [24].

Here reported data raise the intriguing possibility that thyroxin supplementation prior to fertility treatments in women with even only high-normal TSH levels (i.e., sub-clinical hypothyroidism) and certainly in women with overt hypothyroidism, may enhance follicular recruitment and/or growth at small growing follicle stages. This, as an automatic consequence, would lead to larger oocyte and embryos yields with in vitro fertilization (IVF) and, therefore, as one would assume, also to higher cumulative pregnancy rates.

A report by Revelli et al. a number of years ago, however, confirmed these assumptions only partially by demonstrating larger oocyte yields after levothyroxin supplementation in euthyroid fertility patients with thyroid autoimmunity; but pregnancy rates improved only when thyroxin supplementation was combined with acetylsalicylic acid and prednisolone treatments [25].

If confirmed, Revelli’s data in combination with here reported outcomes would suggest that hypothyroidism negatively affects female fertility by reducing FOR, which may be correctable by thyroxin supplementation. Frequently associated autoimmunity and/or abnormalities in inflammatory pathways, however, negatively affect implantation potential or even increase miscarriage risks, both in the literature strongly associated with thyroid disease [21]. Unfortunately, the number of patients who demonstrated thyroid autoimmunity in this study was too small to assess effects of autoimmunity outright on clinical pregnancy and live birth rates. A meta-analysis by Toulis et al., however, supports these conclusions by confirming higher miscarriage rates in euthyroid IVF patients with thyroid autoimmunity [26].

Not everybody agrees, however, with the conclusion that hypothyroidism is associated with poor FOR. Based on a very recent cross-sectional study in Belgium, Polyzos et al. reported that neither functional hypothyroid nor thyroid autoimmunity in their patient population were statistically associated with FOR [27].

Their findings are, however, not only difficult to integrate with here reported findings but also with above cited reports in the literature. Moreover, they, themselves, note that their study design did not preclude significant patient selection biases. In addition, their study failed to statistically adjust outcomes for cofounders. A 2012 meta-analysis, in contrast, supports the contention that supplementation with L-thyroxin improves clinical pregnancy outcomes in association with IVF in women with subclinical hypothyroidism and/or thyroid autoimmunity [28].

Magri et al. in the same year concluded that IVF outcomes were negatively influenced by autoimmune thyroid disease but that keeping TSH <2.5mIU/mL may negate such effects [29]. A Turkish group actually reported increased AMH levels in association with Hashimoto’s thyroiditis, and, therefore, suggested that this autoimmune thyroiditis and polycystic ovary syndrome (PCOS) may share a common etiology [30]. As previously noted, Japanese colleagues also very recently reported an association between elevated TSH and low AMH levels [17], Saglam et al., in contrast, after age-adjustment reported a strong statistical association (*P* = 0.008) between autoimmune thyroid disease and AMH levels [31]. Finally, Magri et al. in a very recent follow up study to above cited 2012 study, not surprisingly reported that the likelihood of poor response to ovarian hyperstimulation with gonadotropins (i.e., of poor FOR) was high in women with low AMH but, apparently, not related to autoimmune thyroid disease; when FOR was considered “good,” autoimmune thyroid disease was, however, associated with decreased response to ovarian stimulation [32]. This, of course, is also an anticipated finding because once FOR falls below a minimal threshold, further effects of thyroid autoimmunity can no longer be expected to be visible.

A principal reason why the here presented study was limited to patients in euthyroid range was, indeed, to avoid cofounders that, unrecognized, may affect study results. Our hypothesis was that in this normal range the respective influences of thyroid function and autoimmunity might be the easiest to dissect since secondary effects on ovarian function, like at very low levels of FOR in Magri’s study [32], would be less likely.

The clarity and statistical power of here reported results, their consistency after adjustments for autoimmunity and age appear to support our hypothesis. Further research on the subject, however, appears urgently needed, considering the very obvious difficulties in differentiating effect of thyroid function and autoimmunity on FOR independently.

Such research, however, as this and above cited studies suggest, has to consider the likelihood that thyroid function and thyroid autoimmunity affect female fertility via different effects and/or pathways. Studies, therefore, have to be designed accordingly, by statistically controlling for distinctively contributing factors, such as FOR, thyroid autoimmunity and age; but also with attention paid to how ovaries are being stimulated, and what other potential cofounders may be present. Considering the multiple cofounders that may play a role, only a multicenter study, involving much larger patient cohorts than are usually available to single IVF centers will, therefore, be able to resolve matters.

## Conclusion

The present study offers solid statistical evidence that thyroid function, at least in euthyroid range, plays a more important role in affecting FOR than thyroid autoimmunity. This finding further suggests that, even within what generally is considered normal euthyroid TSH range, close attention should be paid in infertile women to TSH values.
